# Point-of-care ultrasonography supports for decision-making during a complex mountain rescue operation of 10 h of a trauma patient complicated by multiple cardiac arrests: a case report

**DOI:** 10.1016/j.resplu.2025.101148

**Published:** 2025-10-31

**Authors:** Massimiliano Coha, Fabio Passet, Giulia Roveri, Andrea Carelli, Diego Naso, Christian Bracco, Giacomo Strapazzon

**Affiliations:** a Department of Anesthesia and Intensive Care, Ciriè Hospital, ASL TO4 Torino, Italy; b Corpo Nazionale del Soccorso Alpino e Speleologico – CNSAS, Milano, Italy; c Emergency Medicine Unit, San Luigi Gonzaga University Hospital, Orbassano, Italy; d Institute of Mountain Emergency Medicine, Eurac Research, Bolzano, Italy; e MEU/118, Regional Emergency Medical Service Valle d’Aosta, Aosta, Italy; f Department of Emergency, Ciriè Hospital, ASL TO4 Torino, Italy; g Department of Internal Medicine, S. Croce e Carle Hospital, Cuneo, Italy; h Department of Medicine – DIMED, University of Padua, Padua, Italy

**Keywords:** Cardiac arrest, Trauma, Spinal cord injury, Ultrasound, E-FAST, Point-of-care ultrasonography, Rescue, Remote environment

## Abstract

**Background:**

Trauma care in remote mountain environments presents significant challenges due to low resources and difficult terrain. Point-of-care ultrasonography is a promising tool for decision-making in such settings, though its role in pre-hospital care is not yet routine.

**Case presentation:**

A 72-year-old male sustained a traumatic spinal cord injury during a fall at around 2400 m above sea level. Despite four episodes of cardiac arrest due to autonomic dysfunction, a 10-h rescue operation, and difficult terrain, the patient survived. Point-of-care ultrasonography was used to assess potential causes of cardiac arrest, excluding common conditions like pneumothorax or cardiac tamponade, and to guide management. Autonomic dysfunction due to spinal cord injury was suspected. The patient was treated with intramuscular adrenaline, which stabilized vital signs during transport.

**Conclusions:**

This case describes the complexity of managing a severely polytraumatized patient with cardiac arrest in a remote and austere environment. The use of point-of-care ultrasonography was crucial to reduce the likelihood of common causes of traumatic cardiac arrest, and pointed to spinal shock as the most likely etiology, managed thanks to adapting skills of the medical and rescue team.

## Background

Trauma care in mountain rescue is a situation where environmental, topographical, and logistical factors may limit diagnostic and therapeutic options. When air-rescue support is unavailable, transportation can be prolonged, affecting survival and outcome.^1^ Point-of-care ultrasonography is an approach that can support triage and treatment decisions, but its role in the pre-hospital care is not yet routine.^2^

We report the case of a 72-year-old man who experienced four episodes of cardiac arrest caused by autonomic dysfunction following a traumatic spinal cord injury sustained during a night fall in a remote mountain location at around 2400 m above sea level. Despite severe trauma and episodes of cardiac arrest, and a prolonged 10-h prehospital rescue operation in challenging terrain, the patient survived and had a full cognitive recovery.

This case report describes the complexity of managing a severely polytraumatized patient with cardiac arrest in a remote and austere environment. It highlights the critical role of point-of-care ultrasonography to support decision-making and the successful use of the intramuscular route for adrenaline administration during a complex mountain rescue of 10 h with a patient's favorable outcome.

## Case presentation

**Fig. 1:**
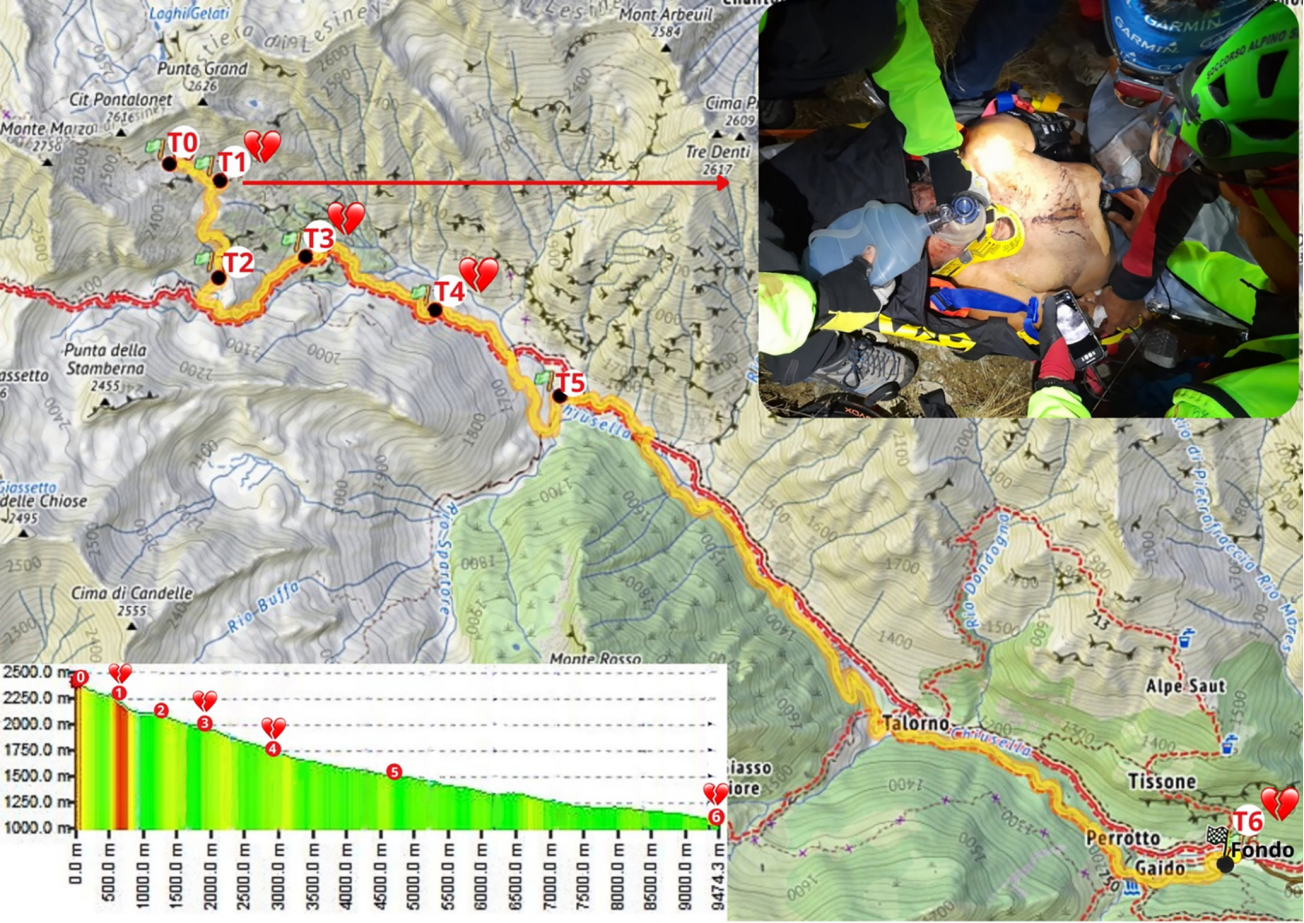
Map showing the timeline and location of the different time points (T0–T6) of the rescue operation. The heart icons indicate the four time points at which cardiac arrest (CA) occurred. T0 (2412 m above sea level): rescue team dispatched to the target point; T1 (2318 m): first cardiac arrest during a verticalization maneuver; T2 (2104 m): missed rendezvous with the helicopter, the patient was conscious; T3 (1956 m): second cardiac arrest; T4 (1756 m): third cardiac arrest, intramuscular adrenaline (0.5 mg) administration was started every 20–30 min; T5 (1567 m): patient transferred to a dirt off-road rescue vehicle; T6 (1072 m): rendezvous with ambulance, fourth cardiac arrest. The lower left box shows altitude above sea level (y-axis) and the distance in meters (x-axis) from the beginning of the rescue (T0) to the point where the patient was loaded into the ambulance (T6). The upper right box shows a photo of the patient undergoing an E-FAST (Extended Focused Assessment with Sonography for Trauma) examination.

A 72-year-old man fell (less than 3 m) at an altitude of around 2400 m in Valchiusella, Italy. The dispatch center was alerted at 7.06 PM (Fig. 1, T0, 2412 m). Helicopter emergency medical services could not be dispatched. Ten mountain rescue technicians and two health-care providers reached the scene, initially using ground vehicles and completing the approach on foot at 11 PM.

During the initial assessment, he had patent airway, adequate ventilation and oxygenation, no signs of massive bleeding, but he had significant disability noted, and multiple facial bone fractures were observed. The patient had a Glasgow Coma Scale (GCS) of 15 but reported cervical pain rated 9 on the Numeric Rating Scale upon palpation, along with reduced upper limb mobility, weakness, and hypoesthesia. The patient was transferred to a stretcher for evacuation, and preventive measures against accidental hypothermia were taken. At 12:15 AM, the first physician arrived on-site and reported no hemodynamic instability. The physician administered 2 g of tranexamic acid intravenously (IV) but no analgesic treatment due to the lack of spontaneous pain. As the terrain was extremely steep, the stretcher was secured with technical rope manoeuvres and human anchors, and there was periodic verticalization during transport. Transport was periodically interrupted to monitor the patient. At 1:50 AM (Fig. 1, T1, 2318 m), the patient experienced sudden clinical deterioration during verticalization. He lost consciousness (GCS 3), developed severe hypotension (blood pressure, BP 70/40 mmHg), and bradycardia (heart rate, HR 45 bpm), followed by cardiac arrest. An Extended Focused Assessment with Sonography for Trauma (E-FAST) and a basic echocardiographic assessment were performed using the Butterfly iQ+ (Butterfly Network, Inc., Guilford, CT, USA) to investigate the underlying cause. The findings revealed neither a relevant amount of free fluid collection in the abdomen and pelvis nor signs of pneumothorax. The heart showed good cardiac contractility, a non-dilated right ventricle, and no evidence of cardiac effusion or tamponade. Advanced life support (ALS) was immediately initiated, including the administration of Adrenaline 1 mg IV, leading to the return of spontaneous circulation (ROSC) after 2 min. The patient quickly regained consciousness (GCS 14: E3 V5 M6). A renewed request for air support was made, but adverse weather conditions (i.e., fog) prevented activation (Fig. 1, T2, 2104 m).

The transport continued by ground rescue. At 3:10 AM (Fig. 1, T3, 1956 m) and at 3:35 AM (Fig. 1, T4, 1756 m), the patient experienced two other episodes of cardiac arrest. Both events were managed with ALS management including the administration of Adrenaline 1 mg IV, and both resulted in ROSC. Based on the clinical presentation suggestive of spinal cord injury (i.e. cervical pain, reduced upper limb mobility, weakness, and hypoesthesia) and the E-FAST findings, autonomic dysfunction was hypothesized to be the main cause of the cardiac arrest. Due to the unavailability of equipment for continuous IV catecholamine infusion, the physician opted for intramuscular (IM) administration of Adrenaline 0.5 mg. This approach successfully maintained stable vital signs during the ground rescue, with additional 0.5 mg IM doses administered whenever the patient’s peripheral pulse became undetectable, with the carotid pulse palpable. At 5:35 AM (T5, 1567 m), the rescue team reached a dirty road and transferred the patient to a dirt off-road rescue vehicle. As the peripheral pulse was not reliably assessable due to the rough terrain and the patient had regained consciousness, Adrenaline administration was discontinued.

**Fig. 2:**
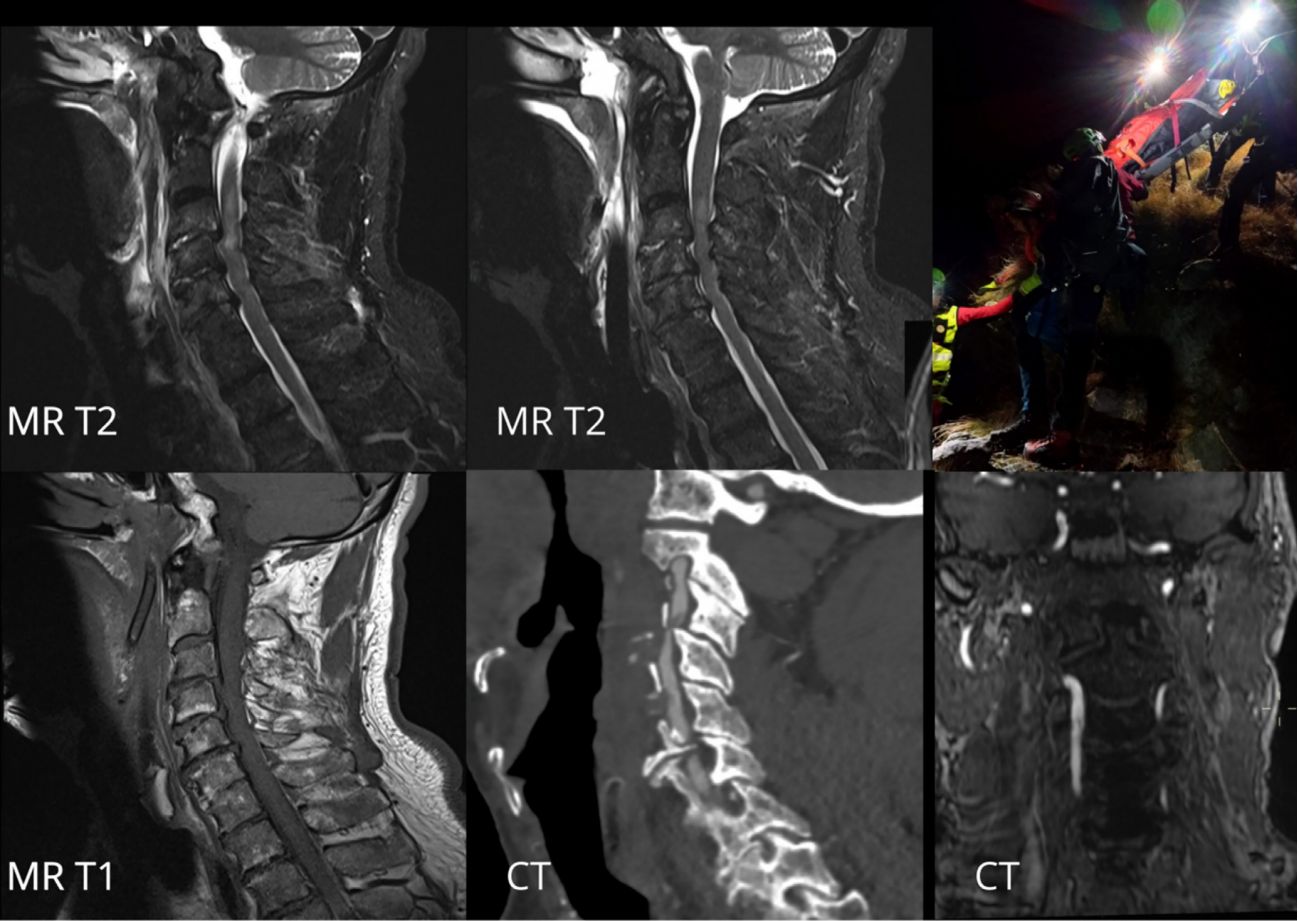
Computed tomography (CT) scan and a magnetic resonance imaging images showing a traumatic spinal cord injury (SCI) at the C2–C4 level, associated with post-traumatic ischemic lesions in the cerebellum secondary to right vertebral artery thrombosis. MR T1 = Magnetic Resonance Imaging, T1-weighted sequence; MR T2 = Magnetic Resonance Imaging, T2-weighted sequence; CT = Computed Tomography.

At 6:00 AM (T6, 1072 m), while being transferred to an advanced medical-transport vehicle, the patient experienced another cardiac arrest. ALS was initiated and ROSC was achieved after administering 1 mg of Adrenaline IV. At 7:00 AM, the patient was admitted to the hospital with a GCS of 13 (E3 V4 M6) with stable hemodynamic parameters. He was spontaneously breathing with a SpO_2_ of 98 %. The patient developed tetraplegia and hypoesthesia/anesthesia below the mammillary line. A total-body computed tomography scan and a magnetic resonance imaging scan (Fig. 2) revealed fractures of the right transverse processes of C1, C2, and C3, retrolisthesis of C3-C4 causing spinal cord compression, as well as right-sided parietal subarachnoid hemorrhage, and multiple fractures of the facial bones. Surgery was performed for the stabilization and decompression of C3-C4, along with orbital fracture fixation. After 23 days in the intensive care unit, he was transferred to the neurology department for further care and rehabilitation. Twenty-four months later, he was able to walk with a rollator, although with some limitations in movement but with a full cognitive recovery.

## Discussion

This case describes environmental, topographical, and logistical factors that limit diagnostic and therapeutic options of a polytraumatized patient during prolong ground rescue in a remote and austere environment. In such a setting, patient outcome ultimately depends on numerous factors, including diagnostic tools like point-of-care ultrasonography and adapting skills of the medical and rescue teams. The patient survived four episodes of cardiac arrest, with autonomic dysfunction resulting from a traumatic spinal cord injury identified as the primary cause. The use of point-of-care ultrasonography reduced the likelihood of more common causes of cardiac arrest, which, along with physical exam, then pointed to spinal shock as the most likely etiology. This then prompted the medical team to tailor therapies for spinal shock. This report also highlights the complexities of performing advanced resuscitation techniques in a remote setting with low resources and significant logistical challenges.

Cardiac arrest following major trauma is typically caused by exsanguination/hypovolemia, hypoxia, tension pneumothorax, or cardiac tamponade.^3^ In this case, point-of-care ultrasonography was crucial for excluding these conditions.^4^ The E-FAST revealed no relevant amount of free fluid in the chest or abdomen and pelvis, no evidence of cardiac tamponade, and no pneumothorax.^5^ Echocardiography showed good cardiac contractility and a non-dilated right ventricle, ruling out both pulmonary and coronary thrombosis as potential causes of cardiac arrest. These findings pointed to autonomic dysfunction, triggered by traumatic spinal cord injury, as the most likely cause.

This case shows the value of ultrasound not only in excluding more common causes of cardiac arrest, but also in guiding the management of a critically ill patient during a prolonged and challenging rescue operation, especially with limited equipment.^6^ Autonomic dysfunction due to traumatic spinal cord injury is a rare but significant phenomenon.^7^ When the spinal cord is injured, particularly at high cervical levels, the sympathetic nervous system may be disrupted, impairing the regulation of heart rate, blood pressure, and vascular tone.^8^ Autonomic dysfunction following is particularly concerning in trauma patients as it can present as unexplained cardiovascular collapse, often confounding the clinical diagnosis and management.^9^, ^10^ In our case, cardiac arrest consistently occurred when the patient was transported in a vertical position on the stretcher. We hypothesize that this position promoted blood pooling in the vasoplegic vessels of the lower body, reducing cardiac preload and output, ultimately leading to cardiac arrest. In our case, the recurrent vasoplegia and bradycardia were treated with repeated administration of Adrenaline 0.5 mg IM. The administration of Adrenaline helped restore vascular tone, increasing the stressed intravascular volume and preventing significant blood pooling in the vertical position.^11^ Intramuscular Adrenaline provided a more gradual and sustained release due to the depot effect, compared to the shorter duration of action of the intravenous route. The approach was based on the similarity between neurogenic shock and anaphylactic shock, both of which are forms of distributive shock.^11^ In anaphylaxis, Adrenaline is commonly administered intramuscularly at a dose of 0.5 mg to restore vascular tone and improve cardiac output.^3^ Applying this principle, and in the absence of an infusion pump for continuous intravenous drug administration, the team applied the same strategy to treat the patient’s neurogenic shock. The repeated doses of intramuscular Adrenaline helped stabilize the patient’s blood pressure and heart rate throughout the prolonged rescue operation, making this approach a novel, yet not evidence-based, aspect in a low-resource setting like a mountain rescue operation without air support.

The management of the patient involved a prolonged and challenging rescue operation lasting over 10 h, with the patient being transported through rugged terrain. The situation was further complicated by the inability to use helicopter-based rescue due to adverse weather conditions, including dense fog. In such a scenario, in terms of temporal length, can be comparable to previous cases described for cave rescue situations.^12^, ^13^ Adapting skills of the medical and rescue teams are fundamental and can be implemented with dedicated training of target hard and soft skills, as especially applied in special rescue situations in mountain rescue or in tactical medicine.^1^, ^13^, ^14^, ^15^

## Conclusions

This case describes the complexity of managing a severely polytraumatized patient with cardiac arrest in a remote and austere environment. The patient survived four episodes of cardiac arrest, with autonomic dysfunction resulting from a traumatic spinal cord injury identified as the primary cause. The use of point-of-care ultrasonography was crucial to reduce the likelihood of exsanguination/hypovolemia, hypoxia, tension pneumothorax, or cardiac tamponade, and pointed to spinal shock as the most likely etiology. This then prompted the medical team to tailor therapies for spinal shock with a successful use of the unconventional intramuscular route for adrenaline administration.

## CRediT authorship contribution statement

**Massimiliano Coha:** Writing – original draft. **Fabio Passet:** Writing – review & editing. **Giulia Roveri:** Writing – original draft. **Andrea Carelli:** Writing – review & editing. **Diego Naso:** Writing – review & editing. **Christian Bracco:** Writing – review & editing. **Giacomo Strapazzon:** Writing – original draft.

## Consent for publication

Written informed consent for publication of clinical data and images was obtained from the patient.

## Funding

No funding was received for this publication.

## Declaration of competing interest

The authors declare that they have no competing interests.
